# Oxidative Modification of Proteins: An Emerging Mechanism of Cell Signaling

**DOI:** 10.3389/fphys.2012.00369

**Published:** 2012-09-14

**Authors:** Stephanie B. Wall, Joo-Yeun Oh, Anne R. Diers, Aimee Landar

**Affiliations:** ^1^Departments of Pathology, University of Alabama at BirminghamBirmingham, AL, USA; ^2^Center for Free Radical Biology, University of Alabama at BirminghamBirmingham, AL, USA; ^3^Department of Biophysics, Redox Biology Program, Medical College of WisconsinMilwaukee, WI, USA

**Keywords:** redox signaling, thiol, 15-deoxy-prostaglandin J_2_ (15d-PGJ_2_), electrophile, reactive species

## Abstract

There are a wide variety of reactive species which can affect cell function, including reactive oxygen, nitrogen, and lipid species. Some are formed endogenously through enzymatic or non-enzymatic pathways, and others are introduced through diet or environmental exposure. Many of these reactive species can interact with biomolecules and can result in oxidative post-translational modification of proteins. It is well documented that some oxidative modifications cause macromolecular damage and cell death. However, a growing body of evidence suggests that certain classes of reactive species initiate cell signaling by reacting with specific side chains of peptide residues without causing cell death. This process is generally termed “redox signaling,” and its role in physiological and pathological processes is a subject of active investigation. This review will give an overview of oxidative protein modification as a mechanism of redox signaling, including types of reactive species and how they modify proteins, examples of modified proteins, and a discussion about the current concepts in this area.

## What are Reactive Species?

“Reactive species” is an umbrella term often used to describe a myriad of small molecules which can participate in chemical reactions. Reactive oxygen species (ROS) include superoxide $\left({\text{O}}_{2}^{•-}\right)$, hydrogen peroxide (H_2_O_2_), hydroxyl radical (HO^•^), and other reactive molecules containing oxygen (Finkel, 2011). Reactive nitrogen species (RNS) are nitrogen-containing molecules including nitric oxide (NO^•^) and higher oxides of NO^•^ such as dinitrogen trioxide (N_2_O_3_), peroxynitrite (ONOO^−^), and nitroxyl anion (HNO^−^; Hill et al., 2010). Reactive lipid species (RLS) are usually derived from unsaturated lipids and include among others, lipid aldehydes (e.g., 4-hydroxynonenal) and reactive prostaglandins of the A- and J-series (Higdon et al., 2012). While these terms cover a wide variety of molecules, they are a convenient, though often vague, way to describe the action of these types of compounds without implying one specific mediator.

Reactive species can be derived exogenously from the diet and environment as well as endogenously through enzymatic or non-enzymatic processes. Exogenous sources of reactive species have been shown to have both beneficial and harmful cellular effects, since they may either participate in cell signaling or may cause macromolecular damage. For example, dietary compounds such as curcumin in curry, and sulforaphane in cruciferous vegetables (e.g., brussel sprouts) are electrophilic and reactive with protein thiols (Brennan and O’Neill, 1998; Hong et al., 2005). These compounds have been shown to have potent anti-inflammatory properties and potential health benefits (Clarke et al., 2008; Lopez-Lazaro, 2008; Zhao et al., 2011). Other bioactive dietary components such as quercetin which is abundant in fruits, and phytoprostanes in fresh vegetable oils have been shown to be metabolized within the cell to reactive species which are anti-inflammatory (Spencer et al., 2003; Karg et al., 2007). On the other hand, compounds such as acrolein in cigarette smoke and acrylamide in fried foods are associated with predominantly deleterious effects such as depletion of cellular glutathione and neurotoxicity (LoPachin and Barber, 2006; Stevens and Maier, 2008).

In addition, there are endogenous sources of reactive species that have specific roles in physiology. For example, nitric oxide synthase (NOS) produces NO^•^ in a controlled manner to regulate the physiological processes of vasodilation (Gruetter et al., 1979), modulation of mitochondrial respiration (Brookes et al., 2002), and immunodefense (Sakiniene et al., 1997). The overall effect of NO^•^ is determined by the amount of NO^•^ produced and the site of production. Low levels of NO^•^, produced by the endothelial isoform of NOS (eNOS), diffuse to adjacent vascular smooth muscle cells to mediate vasodilation. However, high levels of NO^•^ produced by the inducible NOS isoform (iNOS) during inflammation may cause cell death by inhibition of mitochondrial respiration and other mechanisms (Brown and Borutaite, 2002). Other enzymatic sources of reactive species include xanthine oxidase and NADPH oxidases. Additionally, electrons from the mitochondrial respiratory chain can univalently reduce oxygen to generate ${\text{O}}_{2}^{•-}$ (Thannickal and Fanburg, 2000).

Some of the earliest studies in reactive species have centered on ${\text{O}}_{2}^{•-}$, H_2_O_2_, NO^•^, ONOO^−^. H_2_O_2_, in particular, is a potent mediator of signal transduction and can mediate oxidation of protein thiols (Rhee et al., 2003). The cellular actions of H_2_O_2_ are primarily dependent upon the location of production, the concentration of H_2_O_2_, and presence of H_2_O_2_ metabolizing proteins (e.g., catalase, peroxiredoxin, glutathione peroxidase, etc.). The expression of H_2_O_2_ metabolizing proteins is highly regulated with specific isoforms often localized to particular subcellular organelles. Endogenous production of oxidants such as hypochlorous acid (HOCl) and hypobromous acid (HOBr) have also been demonstrated, and their importance in physiology and pathology is becoming more widely recognized since these compounds are thought to play an important role in the antimicrobial response of host immune cells during infection (Weiss et al., 1986; Hurst and Barrette, 1989; Carr et al., 1998).

More recently, the endogenous generation of RLS is emerging an important mechanism of cellular signaling, particularly with respect to inhibiting inflammation, or at higher levels mediating apoptosis. RLS can be derived from oxidation of n-3 or n-6 polyunsaturated lipids which are cleaved from the membrane prior to oxidation. Alternatively, lipids may be oxidized within the membrane giving rise to hydroxy-alkenals and oxidized phospholipids which are active in physiological and/or pathological conditions (Kadl et al., 2004; Catala, 2009). Oxidized lipids can be formed enzymatically (e.g., lipoxygenase, cyclooxygenase), or non-enzymatically via lipid peroxidation or nitration pathways. There are a large number of unique RLS, however, these can typically be grouped according to which functional groups are present. Major classes of reactive lipids include lipid aldehydes, α,β-unsaturated carbonyls, and nitroalkenes [see (Higdon et al., 2012)]. In most cases, reactive lipids are electrophilic and can react with cellular nucleophiles which include certain amino acid side chains. Some reactive lipids contain more than one functional group, which is the case with 4-hydroxynonenal (4-HNE). 4-HNE can participate in Schiff base reactions involving the aldehyde group, and/or Michael addition reactions involving the electrophilic β-carbon [see (Higdon et al., 2012)].

## Redox Signaling or Oxidative Damage?

At the cellular level, the effects of reactive species can range from cell death due to widespread damage to macromolecules to more subtle effects on cell metabolism, morphology, or signaling pathways (Martindale and Holbrook, 2002). The overall impact of a reactive species on cellular function varies with reactive species, but can also be affected by cell type, levels of endogenous antioxidants and antioxidant enzymes, differentiation state, extracellular environment, and many other factors (Jones and Go, 2010). Generally, severe oxidative stresses occur during exposure of cells to radiation, occupational exposures to highly reactive chemicals such as paraquat, and during pathology (Shacter, 2000). In these cases, reactive species can cause DNA base modification, phospholipid damage, and irreversible protein oxidation which can lead to cell death or mutagenesis. However, mild to moderate oxidative stresses occur during normal physiology, exercise, or growth. The reactive species generated during these types of stresses lead to cellular protection, improved metabolism, and resistance to oxidative damage (Higdon et al., 2012).

**Figure 1:**
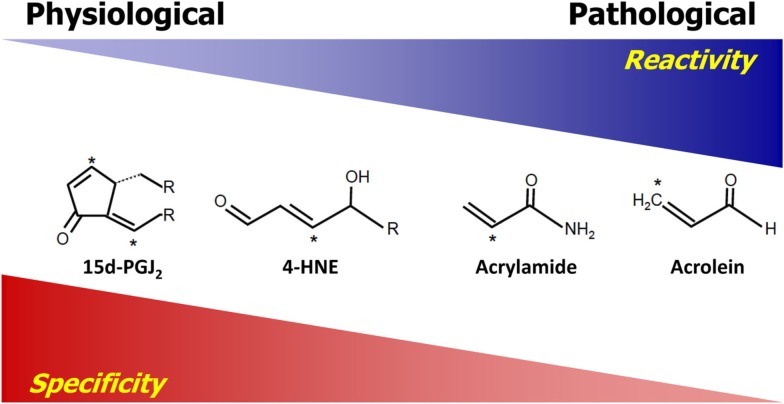
**Reactivity versus specificity**. The compounds shown are electrophilic and have a very wide spectrum of reactivity. They are listed in order of increasing reactivity from left to right. The relative reactivity is predicted based on previously published reaction rate constants and electrophilicity indexes [see (LoPachin et al., 2007; Rudolph and Freeman, 2009)]. Reactivity is inversely correlated with their specificity in that those on the left are more specific for Cys, which is the most reactive nucleophilic residue in the cell. On the structures listed, the asterisk denotes the electrophilic atom(s) of the compound.

The difference between cellular signaling and damage by reactive species are governed by a number of properties inherent to the reactive species. Generally, the relative reactivity and specificity are two major properties dictating how reactive species will interact with targets. For the purpose of this review, the term “reactivity” is used to describe the ability and the rate at which reactive species can chemically react with a target. Highly reactive species, such as hydroxyl and alkoxyl radicals, have relatively high reaction rate constants, which in the case of hydroxyl radical is close to the diffusion limit, and therefore will react with and modify targets which are closest to the site of production of these species with little to no specificity (Sies, 1993). Reactivity is derived from the chemical properties of both the reactive species and the target. The term “specificity” refers to the ability of reactive species to make adducts with one class of molecules preferably over another. Reactivity and specificity have an inverse relationship in that, generally, reactive species which are more reactive are also less specific. Thus, highly reactive species usually cannot achieve specificity. The relationship between the reactivity and specificity of selected electrophilic lipid species (electrophiles) is used as an example of this relationship in Figure 1. The electrophiles are listed in relative order of reactivity with those on the left having the lowest reactivity and those on the right having the highest (LoPachin et al., 2007). Those reactive electrophiles found on the right of the diagram exhibit less specificity in reactions with nucleophilic targets than those on the left. Importantly, highly reactive compounds are also associated with macromolecular damage and tend to form adducts with protein targets which are relatively abundant, since the probability of a reaction with these proteins is higher. Conversely, reactive species with lower reactivity, as exemplified by the compounds on the left side of Figure 1, exhibit more specificity, resulting in the formation of adducts with certain protein residues (e.g., cysteine). As opposed to the highly reactive compounds, these less reactive electrophiles are usually associated with signaling and the modification of a relatively small subset of proteins which is not solely dependent on the abundance of the target and more dependent on the specificity toward a target. We have previously shown that an electrophilic lipid with low reactivity at sublethal doses forms adducts with primarily cysteine residues and alters cell signaling (Levonen et al., 2004; Diers et al., 2010a). Thus, demonstrating that reactive species having low reactivity are more likely to participate in cell signaling than damage (Levonen et al., 2004; Diers et al., 2010a). This concept is also applicable to the reactivity of reactive oxygen and nitrogen species and the relative reactivity of these compounds is reviewed elsewhere (D’Autreaux and Toledano, 2007; Toledo and Augusto, 2012). However, it is important to note that even highly reactive species at low levels may be able to mediate cell signaling, and reactive species with lower reactivity can be damaging at high levels.

Beyond reactivity and specificity, other important factors are involved in determining how reactive species cause specific cellular effects. These include, but are not limited to: (1) site of production, (2) localization, and (3) reactivity of the target. The site of production of the reactive mediator is important with highly reactive species which have limited diffusion distances since these molecules cannot mediate downstream signaling unless the target is nearby (Forman et al., 2004; Pacher et al., 2007). The site of production may also allow for the accumulation of a reactive species, thereby resulting in locally elevated concentrations necessary for signaling. Also, the site of production of those reactive species which are more reactive and therefore less specific is important for dictating a response. Some enzymes produce reactive species in a localized region, such as membrane lipid rafts (Chen et al., 2009). For example, there is evidence that an isoform of the enzyme NADPH oxidase which produces superoxide is located at specialized lipid rafts with other proteins sensitive to superoxide (Jin et al., 2011), thus creating a redox signaling hub. For this reason, it may be speculated that for a reactive species (especially in the case of highly reactive species) to produce a signaling effect, it must either be produced or localized to the “correct” location in order to elicit a specific response. The understanding of location of production of reactive species will most definitely become an increasingly important area of future studies.

Some reactive species are stable enough to diffuse away from the site of production. For example, hydrogen peroxide is relatively stable and can diffuse away from the site of production before reacting (Winterbourn, 2008). Other reactive species such as the electrophilic lipid, 15-deoxy-Δ^12,14^-prostaglandin J_2_ (15d-PGJ_2_) are very stable, and can diffuse between cells within a tissue to mediate the resolution of inflammation (Gilroy et al., 1999). 15d-PGJ_2_ has also been shown to localize to the mitochondrion based on mitochondrial membrane potential (Landar et al., 2006). The reason for this localization is not clear, however, a structurally similar prostaglandin, PGE_2_, which is not reactive does not localize to the mitochondrion, suggesting that the localization is dependent on the electrophilic nature of the molecule (Landar et al., 2006). Interestingly, the effects of 15d-PGJ_2_ within the mitochondrion may be further enhanced by directing the molecule to the desired compartment using molecular targeting strategies. For example, 15d-PGJ_2_ can exhibit protective effects against oxidative stress, however, when this molecule is directed to the mitochondrion by conjugation to a lipophilic cation, its apoptotic signaling is enhanced (Diers et al., 2010b). Since adducts can accumulate over time, the actions of signaling molecules such as 15d-PGJ_2_, which function via oxidative post-translational modifications, are determined by the relative rate of production of the species versus the rate of adduct turnover by the cell (Oh et al., 2008).

Lastly, it is worth mentioning that the reactivity of the target is important in determining the effects of a reactive species. It is well-established that some reactive species can react with and damage DNA by chemically adducting to specific bases (LoPachin and Decaprio, 2005). The next section will focus on some of the specific properties of the nucleophilic protein targets. Therefore, for the purpose of this review, we will focus on oxidative modifications of protein residues by low to moderate levels of reactive species which are commonly encountered in pathology and physiology.

## Examples of Oxidative Post-Translational Modification of Proteins

It is becoming widely appreciated that reactive species produced in a controlled manner can covalently adduct to specific amino acids in order to elicit a cellular effect. The most commonly studied amino acids modified by reactive species are tyrosine and cysteine. The endogenous modification of tyrosine by reactive species in biological systems has been a subject of interest since the discovery of protein nitrotyrosine modifications in human atherosclerosis (Beckmann et al., 1994). Most reports on nitrotyrosine involve the use of this modification as a marker of peroxynitrite or other RNS formation, and suggest that this modification is deleterious. In fact, nitrotyrosine has been associated with the development of a number of pathologies including heart failure, atherosclerosis, aging, and hypertension [for review see (Pacher et al., 2007)]. However, nitrotyrosine may also be involved in physiological processes (Pacher et al., 2007). Whether tyrosine nitration is a normal mechanism of redox signaling or a marker of protein damage is currently an area of active investigation, but there is some evidence that low levels of peroxynitrite may cause modification of specific tyrosine residues which demonstrates that not all tyrosyl residues are equally susceptible to modification (Schmidt et al., 2003).

**Figure 2:**
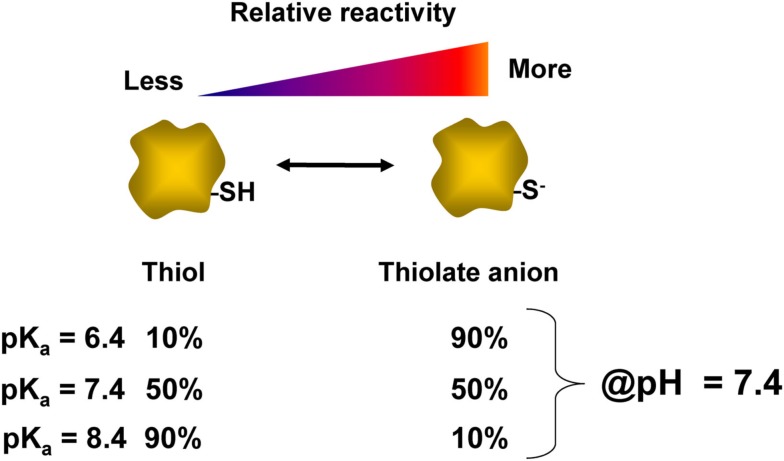
**Relationship of thiol pK_a_ and reactivity**. The acid dissociation constant (pK_a_) of a thiol group determines the ratio of thiol:thiolate at a given pH. This value varies for each cysteinyl thiol and is influenced by the local protein environment. At a physiological pH of 7.4, the relative percentages of protonated thiol and deprotonated thiolate anion are listed for cysteine residues which have different pK_a_s of 6.4, 7.4, or 8.4. The thiol with a pK_a_ of 6.4 is expected to be nearly 90% in the thiolate form at pH 7.4, and is therefore more reactive than thiols with higher pK_a_ values.

Perhaps, the most well characterized residue involved in redox signaling is cysteine. Cysteine accounts for an estimated 1.9% of residues within proteins, and a small number of these cysteines are known to participate in redox signaling (Go et al., 2011). The thiol group (-SH) on the side chain of cysteine can act as a switch for redox signaling and homeostasis. Because the sulfur atom has multiple oxidation states, the side chain of cysteine is readily oxidized to various products, some of which have specialized functions (Jacob et al., 2003). The protonated form of the thiol group (-SH) is not particularly reactive, but the deprotonated form (-S^−^), or thiolate anion, is nucleophilic since it is rich in available electrons (LoPachin et al., 2007). For this reason, not all cysteines have thiols which are equally intrinsically reactive. This inherent reactivity is dictated by a number of contributing factors including accessibility and acid dissociation constant (pK_a_) of the thiol group. The pK_a_ of a thiol is defined as the pH at which 50% of that thiol is in the deprotonated state. Thus, as shown in Figure 2, at physiological pH (7.4) the reactivity of the thiol can vary widely depending on the pK_a_ of the thiol. Thiols which have a relatively low pK_a_ tend to exist in the depronated thiolate anion form, and are thus more likely to be modified by a reactive species. For this reason, the pK_a_ is important in determining the specificity of cysteine modifications by reactive species such as hydrogen peroxide and electrophiles (Rhee et al., 2003; Martyniuk et al., 2011). The thiolate group can participate in reactions with electrophilic reactive species either via nucleophilic substitution reactions or by Michael addition to form covalent adducts. There is also evidence demonstrating site-selective modification of cysteine residues within a single protein by different reactive species (Renedo et al., 2007; Jones, 2010) though characterization of this type of regulation for a broad range of proteins and reactive species has not been reported to date.

The next sections will highlight some specific examples of reactive species modifications organized by type of modification and the functional consequences for each example will be discussed.

### Tyrosine nitration of prostacyclin synthase

Protein tyrosine nitration occurs with the addition of an NO_2_ group to the *ortho* position of the aromatic ring of tyrosine. Tyrosine residues have been shown to be nitrated by at least two multistep mechanisms involving RNS. One pathway involves the formation of a tyrosyl radical which then reacts with a peroxynitrite radical breakdown product, ${\text{NO}}_{2}^{•}$, to produce 3-nitrotyrosine (for reviews see (Pacher et al., 2007; Peluffo and Radi, 2007). Peroxynitrite is a potent oxidant formed by the reaction of superoxide anion with NO^•^ and is produced during inflammatory processes. Interestingly, the main source of superoxide production in the vasculature during inflammation is from NOS itself. During inflammation, the tetrahydrobiopterin (BH_4_) cofactor within the NOS enzyme can become oxidized, thereby uncoupling the enzyme and allowing electrons to reduce molecular oxygen to superoxide. Thus, NOS produces less NO^•^, and ultimately decreases NO^•^ bioavailability (Alp and Channon, 2004). Tyrosine nitration has been of interest during conditions of inflammation by changing the function of target proteins.

Prostaglandin I_2_ synthase (PGIS) is one example of a protein which is modified by tyrosine nitration. PGIS is a heme-containing protein which catalyzes the rearrangement of prostaglandin H_2_ to prostaglandin I_2_ (or prostacyclin). PGIS, also known as CYP8A1, is a member of the cytochrome P450 superfamily and controls vascular tone by regulating the production of prostacyclin in the vasculature. Because prostacyclin is a potent vasodilator, PGIS activity has been shown to play an important role in vascular protection (Wu and Liou, 2005). During times of chronic inflammation and subsequent production of peroxynitrite, the tyrosine residue of PGIS, Tyr430, is nitrated resulting in a decrease in PGIS catalytic activity. This tyrosine nitration, in turn, inhibits prostacyclin-dependent relaxation [for review, see (Zou, 2007)]. Moreover, formation of peroxynitrite also decreases the bioavailability of NO^•^, a key vasodilator, and further promotes vasoconstriction. Though the effects are usually deleterious to vascular function, PGIS represents an example of a protein which is modified by low levels of a reactive species, peroxynitrite (Zou et al., 1999).

### Cysteine oxidation of peroxiredoxin

Oxidation of cysteines is another widely recognized mechanism of redox signaling (Forman et al., 2004; Winterbourn and Hampton, 2008; Paulsen and Carroll, 2010; Finkel, 2011). Disulfide bond formation has long been known to be important in protein structure and function (Tu and Weissman, 2004), and more recently its role in redox signaling has been demonstrated (Frand et al., 2000; Jones et al., 2004). Currently, there is an interest in oxidation of thiols which results in the addition of oxygen(s) to the sulfur group of the amino acid cysteine. The reaction of hydrogen peroxide (H_2_O_2_) with the deprotonated cysteinyl thiol of proteins produces an oxidized thiol or sulfenic acid (R-SOH; Poole et al., 2004). A sulfenic acid may be oxidized again to yield a hyperoxidized sulfinic acid cysteine (R-SO_2_H). With increasing levels of reactive species, cysteines can further be oxidized to a sulfonic acid (R-SO_3_H; Poole et al., 2004). While sulfenic acids are enzymatically reversible by the glutathione and thioredoxin enzyme systems (Berndt et al., 2007), the sulfinic state can only be reversed enzymatically in certain proteins. Sulfonic acid modification is thought to be irreversible and may represent protein damage, rather than signaling.

An example of thiol oxidation is found in the peroxiredoxin (Prx) family of enzymes (Stacey et al., 2012). These proteins contain a reactive cysteinyl thiol in the active site, and the formation of sulfenic acid occurs in their normal catalytic cycle (Rhee et al., 1999, 2005; Stacey et al., 2012). Prxs have the capacity to protect proteins from oxidative damage induced by hydrogen peroxide in a thiol dependent manner. Two cysteine residues, corresponding to Cys47 and Cys170 of yeast Prx are highly conserved. In the presence of hydrogen peroxide, Cys47 gets oxidized to the sulfenic acid intermediate (Cys–SOH). Cys170 is responsible for forming a disulfide linkage with Cys47 (Cys47–S-S–Cys170) in order to resolve the sulfenic acid (Chae et al., 1994). Through a disulfide switching mechanism, the enzyme thioredoxin completes the catalytic cycle by reducing the disulfide bond between Cys47 and Cys170. In addition to H_2_O_2_, Prx directly reduces peroxynitrite and lipid peroxides (Bryk et al., 2000). Importantly, Prx can be hyperoxidized in the presence of excess hydrogen peroxide to the sulfinic acid form which inactivates its function. Sulfinic acid formation in many proteins is not reversible, but sulfinic acid in Prx has been shown to be reduced by a unique thiol dependent enzyme, sulfiredoxin (Chang et al., 2004). Thus, Prx plays a crucial role in redox signaling since it regulates peroxide levels, but in turn is regulated by peroxide itself (Rhee et al., 2012).

### *S*-nitrosation of ryanodine receptor 2

*S*-Nitrosation is the post-translational modification of a thiol group to form an *S*-nitrosothiol which has the general structure of R-S-N = O (Zhang and Hogg, 2005). While the mechanism for RSNO formation in biological systems remains poorly understood, RSNOs are thought to be downstream mediators of nitric oxide signaling. Because of the critical role of nitric oxide in cardiovascular (patho)physiology, much interest has focused on *S*-nitrosation of proteins involved in excitation-contraction coupling in the heart (Gonzalez et al., 2009). In the beating heart, membrane depolarization causes the opening of L-type voltage-gated channels in the plasma membrane and calcium (Ca^2+^) influx into cardiac myocytes. This Ca^2+^ influx then triggers the release of Ca^2+^ from the sarcoplasmic reticulum through the opening of ryanodine receptors, a process termed Ca^2+^-induced Ca^2+^ release (CICR; Fabiato, 1983). Ryanodine receptors as well as other channels involved in CICR (e.g., voltage-gated sodium and potassium channels) have been shown to be targets of *S*-nitrosation (Gonzalez et al., 2009). However, here, we will discuss in detail only the modification of ryanodine receptors.

Ryanodine receptor 2 (RyR2) is the isoform expressed in the heart. The early observation that treatment of RyR2 in reconstituted lipid bilayers with agents which initiate *S*-nitrosation (e.g., *S*-nitroso-*N*-acetyl penicillamine; SNAP) increased the open probability (P_O_) of the channel provided one of the first links between nitric oxide signaling and Ca^2+^ handling at the level of sarcoplasmic reticulum. Moreover, this effect was reversed with the addition of thiol reducing agents, further implicating reversible post-translational modification as a mechanism for regulation of RyR2 (Stoyanovsky et al., 1997). RyR2 has 89 cysteine residues per monomeric subunit, and approximately 21 of these are thought to exist as free thiols. Modification of multiple cysteine residues is required for full activation of RyR2 by *S*-nitrosation (Xu et al., 1998). Cardiac ryanodine receptors have also been shown to be constitutively *S*-nitrosated *in vivo* (Sun et al., 2008), and changes in the redox state of RyR2 thiols have been observed in cardiac pathologies (Yano et al., 2005; Terentyev et al., 2008). Examination of ryanodine receptors isolated from canine heart and rabbit skeletal muscle show that these proteins are susceptible to multiple oxidative modifications including *S*-nitrosation, *S*-gluthionylation, and oxidation to form disulfide bonds, and more recent studies demonstrate that ryanodine receptors co-immunoprecipitate with NOS enzymes, implying that ryanodine receptors are spatially linked to an endogenous source of nitric oxide (Barouch et al., 2002; Martinez-Moreno et al., 2005). Taken together, these studies specifically highlight the important role of *S*-nitrosation in mediating downstream nitric oxide-dependent effects on Ca^2+^ handling in the heart and, more generally, point to a mechanism by which nitric oxide regulates cellular events beyond its canonical role in cGMP-dependent responses.

### Electrophilic lipid modification of Keap1

**Figure 3:**
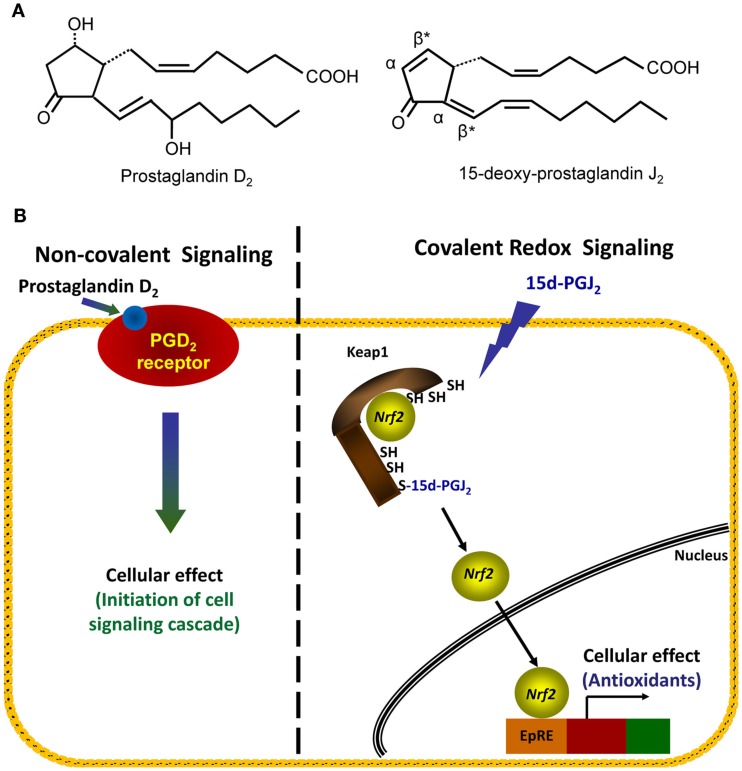
**Comparison of non-covalent and covalent signaling molecules**. **(A)** The structures of two prostaglandins, PGD_2_ (left structure) and 15d-PGJ_2_ (right structure), are shown. Electrophilic carbons which arise from presence of α, β-unsaturated carbonyl functional groups are denoted by asterisks, and α- and β-carbons are indicated. **(B)** Left hand panel: in the non-covalent or classical receptor-ligand model (left hand panel), the ligand PGD_2_ is recognized by a specific receptor on the cell membrane. The binding event causes the activation of a signaling cascade which ultimately changes cellular function. Right hand panel: in the covalent signaling model, a reactive species 15d-PGJ_2_ directly forms a covalent adduct with select proteins, in this case, the cytosolic repressor protein Keap1. This binding event causes changes in endogenous antioxidant protein transcription via the transcription factor Nrf2.

The covalent modification of thiol groups of cysteine residues by electrophiles is another example of an oxidative post-translational modification which results in redox signaling (Higdon et al., 2012). Depending on the type of electrophile, adducts may be formed with nucleophilic cysteinyl thiols through Michael addition or through a nucleophilic substitution (S_N_2) reaction (Hill et al., 2009). Michael addition involves the formation of an adduct which is equal to the exact mass of the electrophile and nucleophile. Nucleophilic substitution results in the formation of an adduct and a leaving group, such as an iodide or chloride moiety from the electrophile. Cyclopentenone prostaglandins, such as 15d-PGJ_2_, are electrophilic lipids which contain an α,β-unsaturated carbonyl group (Figure 3A, right hand panel). During adduct formation, the lipid forms a covalent adduct by Michael addition of the electrophilic carbon with the cysteinyl thiol group of the protein. In the case of 15d-PGJ_2_, there are actually two β-carbons which are electrophilic (Figure 3A right hand panel, denoted by asterisks) due to the presence of two double bonds flanking the carbonyl. However, the β-carbon within the cyclopentenone ring is most commonly found to form adducts with proteins (Uchida and Shibata, 2008). Interestingly, it has been reported that the modification of thiols by electrophilic compounds can be exquisitely site-specific, since different thiols within a single protein can be modified by different cyclopentenone prostaglandins (Gayarre et al., 2005). One well characterized protein modified by electrophilic lipids is the Kelch-like ECH-associated protein 1 (Keap1; Itoh et al., 2004; Levonen et al., 2004), which is involved in the redox regulation of transcription (Brigelius-Flohe and Flohe, 2011).

Keap1 (also known as iNrf2) is normally found in the cellular cytoplasm complexed with the transcription factor Nrf2 (Kaspar et al., 2009). The interaction of Keap1 with Nrf2 under basal conditions directs Nrf2 for degradation by the ubiquitin-proteasomal system (McMahon et al., 2003; Kobayashi et al., 2004). Keap1 cysteine residues, Cys273 and Cys288, have been shown to be modified by 15d-PGJ_2_ thereby preventing proteasomal degradation of Nrf2 (Levonen et al., 2004; Yamamoto et al., 2008). Thus, modification of Keap1 allows for accumulation of Nrf2 and promotes its subsequent translocation to the nucleus (Kobayashi et al., 2006) where Nrf2 regulates the transcription of a number of proteins under the control of the antioxidant response element/electrophile response element (ARE/EpRE) such as glutathione synthase and heme oxygenase-1 (HO-1; Levonen et al., 2004; Wakabayashi et al., 2004). In this way, Keap1 is thought to play a critical role in the cellular response to electrophiles (Dinkova-Kostova et al., 2005). Moreover, activation of Nrf2 by electrophiles through this mechanism and the resulting ARE/EpRE-dependent gene transcription has a major impact on the resolution of inflammation (Levonen et al., 2004).

## Covalent Modification as a Mechanism of Cell Signaling

As illustrated above, the modification of specific amino acids by reactive species can elicit changes in protein function and thereby mediate redox signaling. Covalent redox signaling differs from classical receptor-ligand mediated signaling in several critical ways. This can be illustrated by comparing structurally related endogenous lipids: the non-electrophilic prostaglandin PGD_2_ and the electrophilic prostaglandin 15d-PGJ_2_ (Figure 3B). As a classic receptor-mediated ligand, PGD_2_ binds reversibly to a G-protein coupled receptor and initiates a signaling cascade. In contrast, 15d-PGJ_2_ forms a covalent adduct with its target protein(s) which results in a specific downstream effect (as described above for its effects on the Keap1/Nrf2 system; Figure 3B). The signaling of PGD_2_ and 15d-PGJ_2_, both are dependent on the amount of ligand bound to the “receptor” in order to initiate a signaling cascade. For a non-covalent ligand, such as PGD_2_, this is determined by the concentration of ligand present at a given time. However, due to the ability for 15d-PGJ_2_ to covalently modify its “receptor(s),” adducts may accumulate over time, even at low concentrations of the ligand. Thus, the steady-state concentration of 15d-PGJ_2_ and similar covalent ligands is not necessarily the primary determinant of signaling, but rather the absolute amount of the ligand present over time. For example, we have observed that exposure of endothelial cells *in vitro* to low amounts of a reactive species over a longer period of time will lead to the gradual accumulation of adducts and a level of signaling comparable to an exposure to a higher bolus dose for a shorter period of time (Oh et al., 2008). In this experiment, 20 additions of 0.1 nmol 15d-PGJ_2_ over 7 h resulted in similar induction of HO-1 and GSH as a bolus dose of 2 nmol lipid at t = 0 h in endothelial cells. Thus, covalent redox signaling molecules may result in sustained signaling at very low levels, and do not need to achieve high concentrations in order to be efficacious. We are only now beginning to appreciate the importance of treatment conditions when comparing reactive species across studies.

## Discussion of the Potential for Coordinated Changes in Cellular Function

Another notable characteristic of covalent redox signaling is that, because there is specificity for which proteins are modified, there is often a relatively small group of proteins which are modified simultaneously. Therefore, the downstream effect is likely to be a summation of the effects of the modification of targeted proteins. In addition, covalent redox signaling pathways have been shown to cross-talk with other signaling pathways, such as phosphorylation cascades and calcium mobilization (Diers et al., 2010a; Klomsiri et al., 2011). Thus, the overall activity of a redox signaling pathway will be dependent on which proteins are specifically modified and the interaction of their coordinated effects.

**Figure 4:**
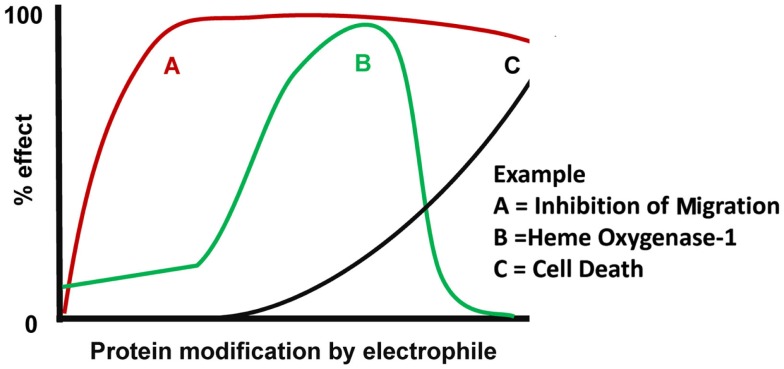
**Model of coordinated cellular effects in response to electrophilic protein modification**. The overall cellular effects of an electrophile are dependent on the susceptibility of different target proteins to be modified by that electrophile. With low levels of electrophile exposure, post-translational modification of the most sensitive target will elicit a cellular response “A.” With moderate electrophile exposure, modification of (an) additional target(s) will result in cellular response “B.” Since response A is still present in this example, the combination of responses A and B cause a coordinated change in cell function by the same electrophilic mediator. With relatively high electrophile exposure, modification of a less sensitive target protein will elicit a new cellular response “C.” Since response A remains and response B is downregulated, the coordinated change in cell function is distinct from that observed at moderate electrophile doses.

Our understanding of how reactive species can modulate different cell functions has been significantly strengthened through the use of biotin-tagged electrophilic lipids (e.g., biotin-15d-PGJ_2_) to evaluate cell function while concomitantly tracking its protein adducts. 15d-PGJ_2_ is particularly interesting since it has been shown to affect cellular functions through pleiotropic mechanisms (Straus and Glass, 2001; Pignatelli et al., 2005). Our studies have shown that 15d-PGJ_2_ reacts with a small group of proteins at low levels, and that with increasing exposure, the number of modified proteins and extent of modification also increases (Oh et al., 2008). This is conceptually illustrated in Figure 4 by a general dose-response of 15d-PGJ_2_ on known cellular effects. The cellular effects which we have observed at relatively low, non-toxic levels include inhibition of migration and cytoskeletal alterations (Diers et al., 2010a). With progressively increasing levels of 15d-PGJ_2_ modification, up-regulation of HO-1 was observed due to the activation of Keap1/Nrf2 pathway, followed by cell death at the highest levels of lipid (Ricart et al., 2009). Interestingly, at the highest levels of 15d-PGJ_2_, HO-1 levels decrease though Keap1 is modified (Ricart et al., 2009), and the reason for this effect remains to be elucidated. However, it is clear that the overall cellular response cannot be explained by the modification of one target protein alone, and is likely due to the modification of multiple proteins. Importantly, the concepts discussed regarding 15d-PGJ_2_ should be applicable to other reactive species which may have signaling roles. Importantly, these concepts of redox signaling by reactive lipid, oxygen, and nitrogen species apply to all cell types, but may vary from one cell type to another based on the differences in protein composition and antioxidant pathways specific to that cell type. Appreciating the complexity of these redox signaling systems and understanding how these pathways are dysfunctional during pathology is of utmost importance to this growing field.

## Conclusion

As more is learned about oxidative post-translational modifications and redox signaling, we will be able to better piece together ways in which individually targeted modifications form protein networks and orchestrate cellular responses. This will obviously be difficult to study experimentally, but is nevertheless important to understand how a given reactive species may have pleiotropic mechanisms and seemingly incongruent effects on cells, ranging from protection against cell death to induction of apoptosis. There is an urgent need for research strategies which take into account systems biology and embrace the complexity of multiple pathways in order to advance new breakthroughs for drug development. In this vein, the inherent properties of a reactive species can be used to direct the covalent modification of a group of proteins in order to elicit specific cellular responses. In this way, novel redox therapeutics can be designed to generate a sustained and defined cellular effect in a variety of pathological conditions.

## Conflict of Interest Statement

The authors declare that the research was conducted in the absence of any commercial or financial relationships that could be construed as a potential conflict of interest.

## Abbreviations

${\text{O}}_{2}^{•-}$, superoxide; 15d-PGJ_2_, 15-deoxy-Δ^12,14^-prostaglandin J_2_; 4-HNE, 4-hydroxynonenal; ARE/EpRE, antioxidant response element/electrophile response element; BH_4_, tetrahydrobiopterin; CICR, Ca^2+^-induced Ca^2+^ release; H_2_O_2_, hydrogen peroxide; HNO^−^, nitroxyl anion; HO^•^, hydroxyl radical; HO-1, heme oxygenase-1; Keap1; iNrf2, Kelch-like ECH-associated protein 1; N_2_O_3_, dinitrogen trioxide; NO^−^, nitric oxide; NOS, nitric oxide synthase; ONOO^−^, peroxynitrite; PGA_1_, prostaglandin A_1_; PGD_2_, prostaglandin D_2_; PGIS, prostaglandin I_2_ synthase; Prx, peroxiredoxin; RLS, reactive lipid species; RNS, reactive nitrogen species; ROS, reactive oxygen species; RyR2, ryanodine receptor 2; SNAP, S-nitroso-*N*-acetyl penicillamine.
